# Deformations in Cement Pastes during Capillary Imbibition and Their Relation to Water and Isopropanol as Imbibing Liquids

**DOI:** 10.3390/ma15010036

**Published:** 2021-12-21

**Authors:** Natalia Mariel Alderete, Arn Mignon, Katrin Schollbach, Yury Villagrán-Zaccardi

**Affiliations:** 1 Magnel-Vandepitte Laboratory, Department of Structural Engineering and Building Materials, Faculty of Engineering and Architecture, Ghent University, Technologiepark Zwijnaarde 60, B-9052 Ghent, Belgium; yury.villagranzaccardi@ugent.be; 2 Smart Polymeric Biomaterials, Biomaterials and Tissue Engineering Research Group, Campus Group T, KU Leuven, Andreas Vesaliusstraat 13, 3000 Leuven, Belgium; arn.mignon@kuleuven.be; 3 Department of the Built Environment, Eindhoven University of Technology, 5612 AP Eindhoven, The Netherlands; K.Schollbach@tue.nl

**Keywords:** capillary imbibition, deformations, C-S-H

## Abstract

The traditional approach for evaluating capillary imbibition, which describes the phenomena as a linear relationship between mass gain and the square root of time, considers a rigid pore structure. The common deviation from the linearity when using the square-root law (manifested in a downward curvature, i.e., slower water ingress) can be explained by considering a changing pore structure during the process caused by the swelling of calcium silicate hydrate (C-S-H) during water ingress. Analysing how the combination of deforming phase (C-S-H), non-deforming phase, and porosity affects the capillary water ingress rate is relevant for a deeper understanding of concrete durability. In this research, the C-S-H content was quantified by means of XRD diffraction coupled with Rietveld + PONKCS, dynamic water sorption (DVS), and SEM/BSE images coupled with phase mapping using PhAse Recognition and Characterization (PARC) software. The porosity was assessed by mercury intrusion porosimetry, water absorption under vacuum, and DVS. Furthermore, to assess deformations occurring with water and a non-aqueous imbibant, capillary imbibition tests with water and isopropanol as invading liquids were performed along with simultaneous deformation measurements. The relation between the relative C-S-H content and porosity has a great impact on the transport process. Samples exposed to isopropanol presented a much larger liquid uptake but significantly fewer deformations in comparison to imbibition with water. The effects of the changing pore structure were also evaluated with the Thomas and Jennings model, from which calculations indicated that pore shrink during imbibition. A comprehensive description of the relation between deformations and capillary imbibition in cement pastes reveals that liquid ingress is highly influenced by deformations.

## 1. Introduction

Concrete in service is far more often in the unsaturated than the saturated state. The most common unsaturated flow is capillary water ingress, and it eventually leads to durability problems during the service life. Many deterioration mechanisms in cementitious materials are largely mediated by water (namely ingress of harmful substances such as sulfates, acids, and chlorides). Hence, a deeper understanding of the transport phenomena can help to enhance the sustainability of concrete structures by accurately describing the ability of concrete to absorb water by capillarity. The most widely used approach to evaluate capillary imbibition in cementitious materials assumes a rigid pore structure and a linear variation of the sample mass gain with the square root of time. The experiment consists of measuring samples gravimetrically at certain time intervals, whereby the mass gain is conventionally plotted versus the square root of time (generally in g·m^−2^·s^−0.5^). This simple experiment is valuable to evaluate the pore structure and durability of cementitious materials. Still, reports generally indicate an ‘anomalous’ lack of linearity [1,2,3,4,5,6,7,8,9], translated in a downward curvature in the g·m^−2^·s^−0.5^ plots. This anomaly is particularly pronounced when experiments last more than 6 h (explicitly addressed for example by the ASTM C1585 by differentiating between an initial and final water ingress rate). The reason for this deviation may be the assumption that the liquid advances sharply through a rigid pore structure. Firstly, the advancing front can only be considered perfectly sharp if all pores have the same size. If the pore size distribution is wide, the time it takes to saturate a certain pore volume becomes different for each pore size. This results in a variation of the shape of the waterfront in cementitious materials contributing to the anomaly. Such variation has been depicted by neutron radiographs [10,11,12]. Secondly, previous research has reported deformations during imbibition in mortar and concrete [13]. This deforming pore structure has a consequential delaying effect on the water uptake evolution. That effect can be modelled with the fourth root of time considering a variable hydraulic diffusivity, as proposed in [9] and then improved in [13]. Such description of the water transport indicates that the pore structure of cementitious materials varies synchronically with water ingress, but additional research can comprehensively connect the deformation and the water imbibition.

Valuable research has described the C-S-H structure and provided information to model it [14,15,16]. Still, there are missing connections between humidity changes in the C-S-H structure and the transport mechanisms at the macroscale. Our proposal that volumetric changes during imbibition are caused by the ability of C-S-H to retain water with consequent swelling requires such connections. C-S-H deformations hinder the water flow causing the mentioned lack of linearity with the square root of time. In fact, this can be addressed not as an anomaly but instead as an intrinsic characteristic of cementitious materials which is generally overlooked when analysing water imbibition. For example, it is well established that volumetric variations occur upon long-term drying shrinkage [17,18,19,20]. The counterpart, swelling, is manifested in the capillary imbibition experiment by a water ingress rate that decreases over the square root of time. There is still a gap in the literature about the effect of different components of the cementitious matrix and the deformations during water ingress. This research aims to explain the link between the volumetric strain and water sensitivity in cement pastes.

Thomas and Jennings [21] proposed a model (TJ model from now on) separating the cementitious matrix into three phases: deformable phase (namely C-S-H when considering water as the deformation driver), porosity, and non-deformable phase (rest of the paste). Such a model makes it possible to evaluate the deformation of a composite considering different degrees of restraint, from a completely free case (no restraints) to a fully restrained case. For the case of cementitious materials, results will be affected by the presence of non-deformable solid phases that enforce a certain internal degree of restraint. With the elegant and simple math of the TJ model, it is possible to determine whether deformations of C-S-H cause expansion or shrinkage of pores.

The present paper aims to analyse the influence of the three mentioned phases (C-S-H, porosity, and rest) on the deformations during capillary imbibition. For that, deformations during imbibition were continuously monitored in cement pastes (with water to binder ratio 0.4 and 0.6) for 28 days. Furthermore, different experimental techniques were used to quantify the porosity and C-S-H content of the pastes. Porosity was measured by mercury intrusion porosimetry (MIP), open porosity by water absorption under vacuum (WA), and dynamic vapour sorption (DVS). An estimation of the porosity was also calculated using the model of Powers and Brownyard (PB). The C-S-H content was determined by X-ray diffraction (XRD) using an internal standard and Rietveld combined with Partial Or Not Known Crystal Structures (PONKCS) analyses [22] and by scanning electron microscopy with backscattered electron (SEM/BSE) images coupled with phase mapping using PhAse Recognition and Characterization (PARC) software. Two imbibing liquids: water (W) and isopropanol (IPA) were used to determine their influence on the deformations and the ingress rate. The difference in deformations for different pastes and the imbibing liquid was analysed with the TJ model.

## 2. Materials and Samples

Paste mixes with water-to-binder-ratios equal to 0.4 and 0.6 (P4 and P6, respectively) were produced with CEM I 52.5 N (Holcim, Mons, Belgium) as a binder. The chemical composition of the cement is given in Table 1. Mixes were cast into prismatic moulds of 40 mm length, 10 mm width, and 15 mm height. Immediately after casting, specimens were wrapped in plastic while maintaining them at 20 °C. Samples were demoulded after 48 h and taken to a conditioned room at 20 °C and >95% relative humidity (RH) until seven days of age when they were preconditioned for the different experiments.

## 3. C-S-H Content and Porosity Determined by Different Experimental Techniques

Significant challenges are faced when determining C-S-H content because of its amorphous structure and indefinite composition [23]. Because of that, for both experimental techniques and hydration models, there are some assumptions necessary to quantify the C-S-H amount. For this research, three experimental techniques were used to compute the amount of C-S-H in cement samples: (1) XRD diffraction coupled with Rietveld + PONKCS analyses [22] using ZnO as internal standard, (2) SEM/BSE images coupled with phase mapping using PARC Software, and (3) sorption measurements of DVS. 

For the case of the XRD analysis, specimen hydration was stopped after seven days following the RILEM recommendations [24]. Half of a prismatic specimen was crushed with a hammer and the internal freshly crushed pieces were collected. Such particles were then ground with mortar and pestle to pass a 1 mm sieve. The whole crushing and sieving procedure was performed as fast as possible (<10 min) to minimise the exposure to air of the sample. Immediately after, the obtained powder was immersed in isopropanol (to stop hydration by solvent exchange). The suspension was filtered using a Buchner filter and flask (filter paper with a pore size smaller than 2 μm). The filter paper was placed on a watch glass and dried for 8 min in a ventilated oven at 40 °C. Afterwards, all samples were further ground to particle size < 63 µm while blended with 10% ZnO as internal standard. For the sample compaction side loading was chosen to prevent preferential orientation of crystals. XRD measurements were taken using CuKα radiation on a Thermo Scientific ARL X’tra diffractometer + Peltier cooled detector operated at 30 mA and 40 kV. Measurements were made at room temperature in step-scan mode (0.02°/seg), scanning the 2θ angle from 5° to 70° to carry out quantitative analyses of the unhydrated cement and pastes. All cement paste samples were tested within 48 h after hydration stoppage.

The PONKCS method addressed the fact that the contribution of partially amorphous phases to the measured diffractogram is proportional to their relative content. The procedure requires a proper model for this contribution and a calibration factor obtained from the pseudo-quantification of a known crystalline phase (i.e., measurements of homogenized samples containing a known amount of ZnO) [22]. The diffuse scattering signal of C-S-H (humps) was fitted using characteristic sets of pseudo-Voigt peaks obtained from a pure well-hydrated sample. Both XRD patterns of P4 and P6 are included in the Supplementary Materials. Quantifications in wt.% were converted into vol.% by considering the densities for the C-S-H (2.6 g/cm^3^ [25]), and pastes (obtained from the water absorption under vacuum) P4 and P6 (1.87 g/cm^3^ and 1.75 g/cm^3^, respectively). 

**Figure 1:**
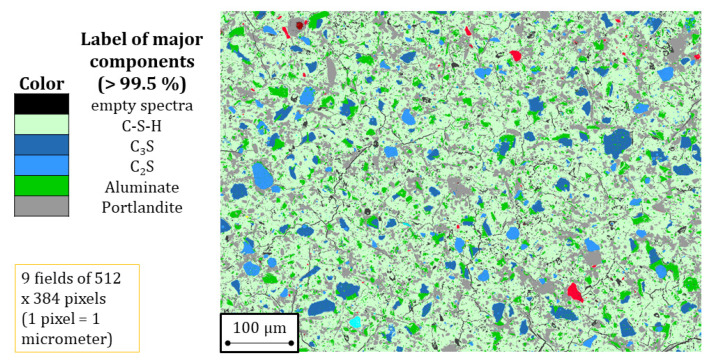
Schematic visualization of PARC results.

The samples for the SEM/BSE + PARC analysis were crushed particles of 5–10 mm size from half a prismatic sample that were impregnated with epoxy resin, polished with an alcohol-based suspension, and carbon-coated. For each individual measurement, 3 × 3 spectral imaging (SI) fields were measured (with one field having a size of 512 pixels × 384 pixels). Each pixel is exactly 1 μm. Since duplicates measurements were made, the total inspected area is 3.54 mm^2^. This is a large enough surface to provide representative results for pastes. The SEM/BSE measurements were performed with a JEOL JSM-7001F SEM with two 30 mm^2^ SDD detectors (Thermo Fisher Scientific, Waltham, MA, USA) and NORAN-System7 hardware with NSS.3.3 software. The accelerating voltage was 15 kV with a beam current of 6.2 nA. The measured SI fields were then analysed with PARC software [26,27]. This software is able to sort the individual pixel spectra into groups based on which element peaks are above a user-defined value. Phase area% are obtained based on the amount of pixels in each group, and can also be represented as a phase map. Phase compositions are based on the quantification of the sum spectra of all pixels from such a group. Considering stereological principles [28,29] and taking into account that the sections analysed were large enough to be statistically representative, the average surface area% can be considered to be the same as the average volume fraction. For this study, the following groups/phases were defined and quantified: unreacted cement (including C_3_S, C_2_S, and aluminates), portlandite, and C-S-H. Only the results for C-S-H are discussed in the paper. A schematic visualization of the PARC software results is provided in Figure 1. All the phase maps obtained from the SEM/BSE images using the PARC software are included in the Supplementary Materials.

For the DVS tests, half of a prismatic sample was ground and sieved into particles between 500 and 1000 μm. Such a particle size is considered to offer the best balance between test duration and carbonation exposure time during grinding [30]. Particle size smaller than 500 μm could generate artifacts while particles larger than 1000 μm significantly increase the time needed to reach equilibrium during DVS [31,32]. Immediately after being ground and until testing, samples were stored in sealed containers in the presence of soda lime to avoid carbonation. Samples were tested in a device set at 20 °C and with a rate of dm/dt < 0.002 wt.%/min as detection limit to continue to the following RH level. Due to the high repeatability of the test, one sample per cement paste mix was tested. The RH levels at which samples were subsequently equilibrated were (98–90–80–70–60–50–40–30–20–10–5–0)% RH. From the data collected of DVS experiments, the C-S-H amount was computed as the ratio of the water amount adsorbed by the paste at RH = 20% (as performed in [30]) to the water amount adsorbed by the C-S-H at RH = 22.8% as determined in [23,31]. Results were transformed into a volumetric fraction (vol.%) considering the density of the samples as obtained from the WA experiments described below.

The porosity of the cement samples was determined by MIP, WA, and DVS. An estimation of the porosity was calculated using the PB model [33]. All porosity results were transformed into a volumetric fraction (vol.%) considering the bulk volume of the samples as obtained from the water absorption under the vacuum experiment described below.

MIP experiments were carried out on gently crushed samples of about 1–2 g. Such samples were obtained from the core of half of a prismatic sample, and the hydration was stopped by solvent exchange with IPA in the same manner as explained before. Then samples were kept under vacuum (0.1 bar) for at least seven days before the test was performed. The MIP maximum pressure was limited to 200 MPa to avoid cracking induced by the pressure [34]. The surface tension and contact angle adopted were 482 mN/m and 142° [35], respectively. The theoretical simplified model of cylindrical pores was used to translate the results into pore entry diameter upon the application of the Lucas–Washburn equation. With the data obtained the intrudable porosity was calculated. For each paste mix, three repetitions were made. 

From the DVS results, the Barret, Joyner, and Halenda (BJH) method [36] was used for the calculation of the pore size distribution in the mesopore range. The Dubinin- Radushkevich (DR) equation [37] was used to calculate the pore size distribution in the micropore range. The results shown as ‘DVS porosity’ are the sum of the calculated mesopores and micropores. 

To determine the water-accessible porosity, by means of the WA, three prismatic samples were cut into slices of (10 ± 2) mm (i.e., *n* = 9 for each cement paste mix). Such samples were put under vacuum (0.1 bar) for 2 h. Afterwards, water was drawn into the vacuum chamber until the sample became fully immersed. The vacuum was released and samples were kept under immersion for 24 h, and then the saturated mass (msat) and immersed mass (mim) were registered. The dry mass (md) was determined after putting the samples in an oven at 50 °C and until stable mass (dm/dt < 0.1 wt.%/24 h). The value of the WA was determined as (msat-md)/(msat-mim). The bulk volume was calculated as (msat-mim), resulting in 1.87 g/cm^3^ and 1.75 g/cm^3^ for P4 and P6, respectively.

The PB model [33] allows studying the formation of cement paste when water and cement are mixed. From this model, the composition of a hydrated paste can be obtained from equations that consider the variation of the components as a function of the hydration degree. For this research, the porosity was also calculated with this model. The degree of hydration was calculated in relation to the non-evaporable (combined) water [38]. The samples, of approximately 0.5 cm^3^, were first dried at 105 °C until the weight loss was lower than 0.1% in a 24 h period. Then, samples were ignited at 1000 °C. The non-evaporable water content was determined as the relative mass loss between 105 °C and 1000 °C (corrected considering the loss on ignition of the cement itself). Considering the value of the nonevaporable water content for a fully hydrated pure cement sample to be equal to 0.24 g H_2_O/g cement [39], the degree of hydration was calculated as the ratio between the assessed non evaporable water content and the non-evaporable water content at full hydration. This allowed calculation of the volumetric fractions of pores (unreacted water), unreacted cement, and hydrated cement paste, as described in [40]. The calculated volumetric fraction of the porosity was used in this study.

## 4. Experimental Set-Up for Capillary Imbibition with Continuous Deformation Measurements

As imbibing liquids water and IPA (purity > 98%) were used. Both were conditioned at 20 °C for 24 h before the beginning of the experiment. The density, the viscosity, and the surface tension of water and IPA were assumed to be equal to 0.9982 g·cm^−3^, 1.002 mPa·s and 72.74 mN·m^−1^, and 0.781 g·cm^−3^, 2.023 mPa·s and 20.9 mN·m^−1^, respectively, at 20 °C [41]. Five samples per liquid and per mix were tested. 

At seven days of hydration, samples were removed from the conditioned room and prepared for the measuring of the volumetric deformations during capillary imbibition. Although the samples were tested at seven days, and hydration could further progress during the imbibition experiment, the influence of such a process on the imbibition experiment is negligible. Many authors [1,2,3] have indicated that the main changes are induced by the interaction between incoming water and C-S-H. Even in totally hydrated concrete cured underwater for 26 years [4] significant decrease in water permeability was found, showing that the continued hydration of clinkers is not responsible for changes during water ingress.

**Figure 2:**
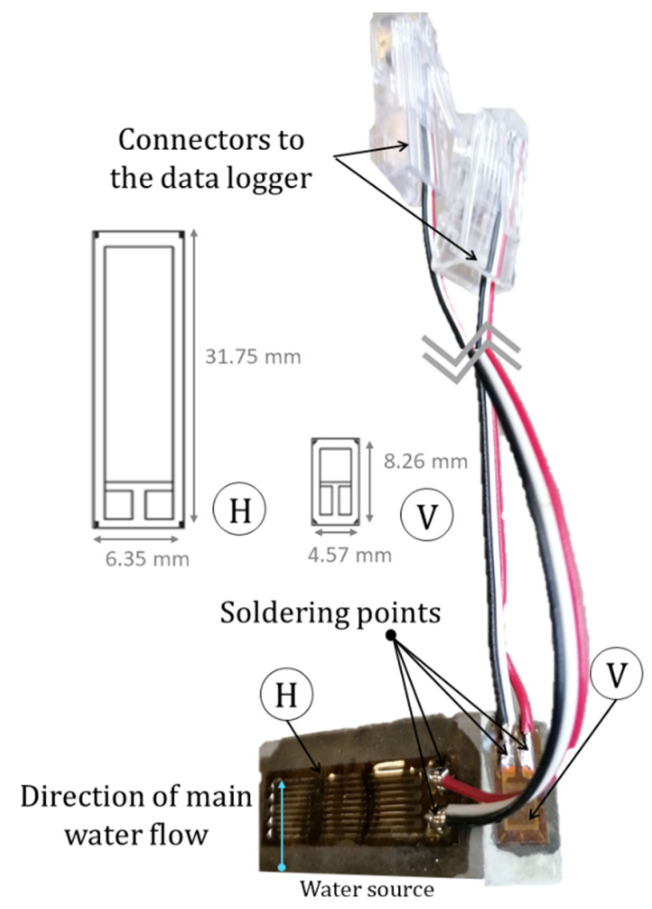
Paste specimen with the attached strain gauges and soldered cable connections (H = horizontal strain gauge, V = vertical strain gauge).

To analyse the vertical and horizontal deformation caused by the water progressing vertically, two strain gauges per sample were applied perpendicular to each other: one placed horizontally (H) and one placed vertically (V) (Figure 2). Strain gauges with linear patterns for use on cementitious surfaces were used, their overall width and height were 6.35 mm × 31.75 mm and 4.57 mm × 8.26 mm, for the horizontal and vertical strain gauges, respectively. The strain gauges were glued to the specimen and then cables were attached with two soldering points. Such cables had a connector to be able to easily detach and attach the samples before and after the weighting of the sample.

After strain gauges were glued, samples were laterally covered with epoxy leaving the top and bottom of the sample uncovered. This allowed one-directional liquid flow and protected the strain gauges connections from direct contact with the imbibant. The preconditioning of the specimens consisted of immersing them in water for 72 h and then drying them in an oven at 40 °C until the weight loss was ≤0.2% in a 24 h period, which took around 1.5 weeks. Then samples were stored sealed in double plastic foil and left at 20 °C for a week to achieve a homogenous moisture distribution.

**Figure 3:**
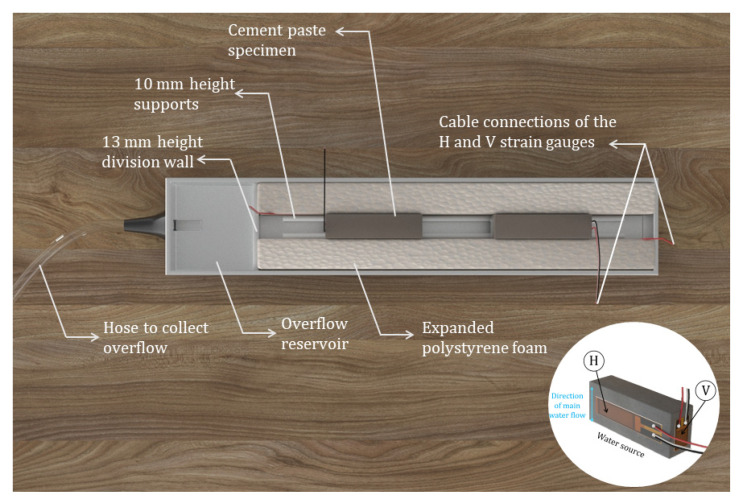
Set-up for capillary imbibition with continuous deformation monitoring.

The set-up for capillary imbibition consisted of aluminium trays with a ‘dam system’ with two separate reservoirs: one for the specimens and the other one for liquid overflow. A small division barrier between such reservoirs maintained a constant immersion level of the specimens. This was materialized by 10 mm height specimen supports and a 13 mm division wall (see Figure 3). This height difference allowed to have the specimens at a constant 3 mm immersion depth. The liquid was permanently refilled and any additional volume over 3 mm was continuously discharged in the reservoir. Two prismatic pieces of expanded polystyrene foam were cut and placed on the sides of the specimen to make sure they kept their position during the experiment, while the flexible nature of such foam prevented any external restraint to the deformation due to the imbibition process.

Together with the continuous deformation recordings, gravimetric measurements were carried out after 0.5 h, 1 h, 2 h, 3 h, 4 h, 5 h, 6 h, and 24 h. After that, samples were measured every 24 h during the first week and every week for a total period of 28 days. When performing long-term capillary imbibition experiments, two marked stages can be related to the change in the main driving force, from capillarity to diffusion and a transition period in between [42]. The primary imbibition period occurs under the main action of capillary forces until moisture is observed on the surface of the upper face of the sample (i.e., wet front reaching the maximum capillary rise). The results were plotted as the capillary imbibition capacity in mg/mm^2^ versus the fourth root of time in s^0.25^. The primary capillary imbibition rate (CIR) was calculated as the slope of the least square fitting line during the primary imbibition period (until the liquid reached the top of the sample).

## 5. Results

The TJ model requires the input of the amount of the deformable phases, amount of non-deformable phases, and porosity. In the cementitious matrix, the deformable phase upon contact with water was represented by the C-S-H content. The amount of non-deformable phase was considered as the remaining solid part of the paste.

### 5.1. C-S-H Content

**Figure 4:**
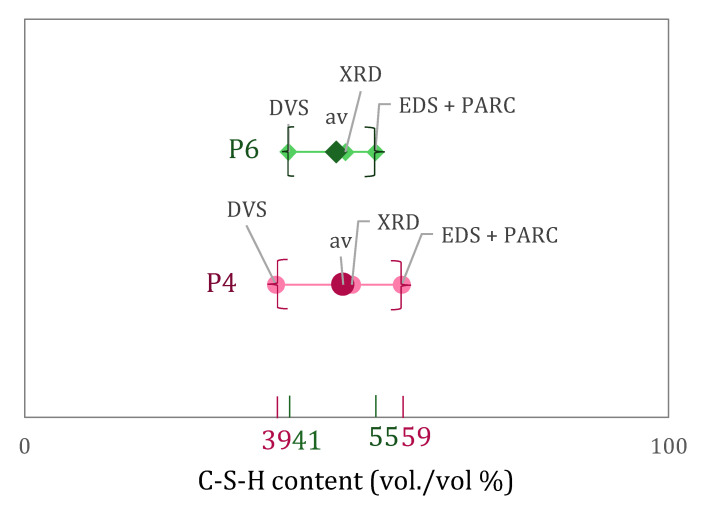
C-S-H content as determined by XRD coupled with Rietveld + PONKCS, by EDS coupled with PARC and by DVS data.

The results of C-S-H content in vol.% from XRD + Rietveld, DVS, and EDS + PARC for P4 and P6 are indicated in Figure 4. All these powerful techniques provide useful information but they all have limitations, and hence the average of the results of the different experimental techniques is given (and used for further calculations) as a way to compute the C-S-H content considering the average and not only one single experiment. Similar C-S-H contents were obtained for both pastes, with slightly higher values for P4. When comparing the results from the different techniques, EDS + PARC gives the largest C-S-H content for both pastes. Some overestimation is possible with this method that cannot differentiate some Afm/Aft inserted in the C-S-H phase. Such phases are finely intergrown and impossible to separate with the EDS resolution of 1 μm. This also means that porosity at or below 1 μm is included in the amount of C-S-H, which is not the case for the other methods. The theoretical C-S-H content calculated from the DVS sorption data seems to give lower results than the average, although values are similar as reported in [30,31].

### 5.2. Porosity

**Figure 5:**
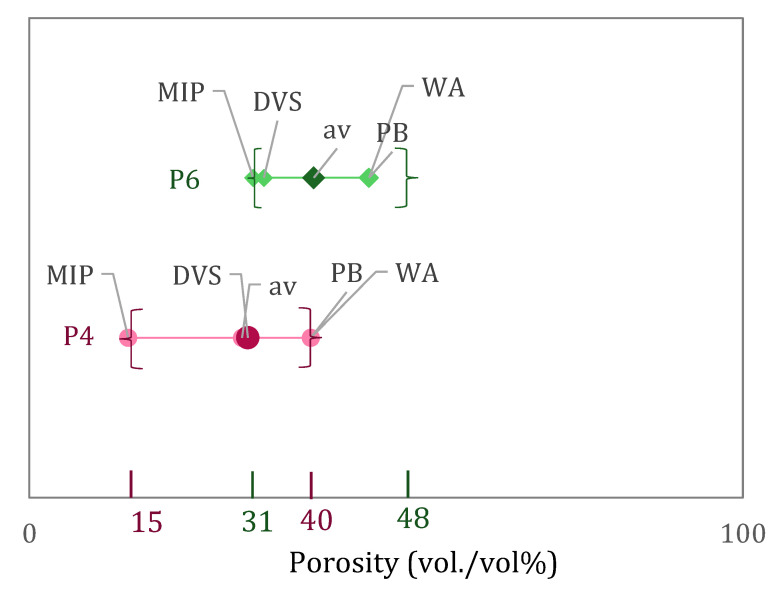
Porosity values obtained from MIP, DVS, WA and PB of pastes P4 and P6.

The porosity values obtained from MIP, DVS, WA, and PB are indicated in Figure 5. As indicated for the C-S-H content results, the average of the results of the different experimental techniques is given as a way to assess the porosity considering the average value and not only one single experiment. However, in this case, it should be noted that each technique does not cover the same pore size range, and this can provide a partial explanation for the differences in the obtained values. Results of intrudable porosity from MIP can go from 0.01 µm to 100 µm while from DVS (combining BJH + DR) much smaller pores are assessed, going from 0.001 µm to 0.05 µm. From WA, as water is drawn into the specimen while being under vacuum, we can expect to be measuring air voids, capillary pores and even going down to the micrometric scale. Results from PB derive from a theoretical calculation where the sum of gel and capillary pores is considered. Despite the differences, these techniques can describe the pore structure and are currently widely used to measure the porosity of cement pastes. 

As it is well-known, the initial water-to-cement ratio influences the final pore content of the material. This effect is seen in the differences between the mixes P4 and P6, in agreement with their corresponding water-to-binder ratios [43]. Results from all techniques resulted in lower porosity for P4 than P6. It was also found that for both P4 and P6 MIP and DVS results provide lower porosity values than WA and PB. This is because WA and PB are able to evaluate a larger pore range than MIP and DVS.

**Figure 6:**
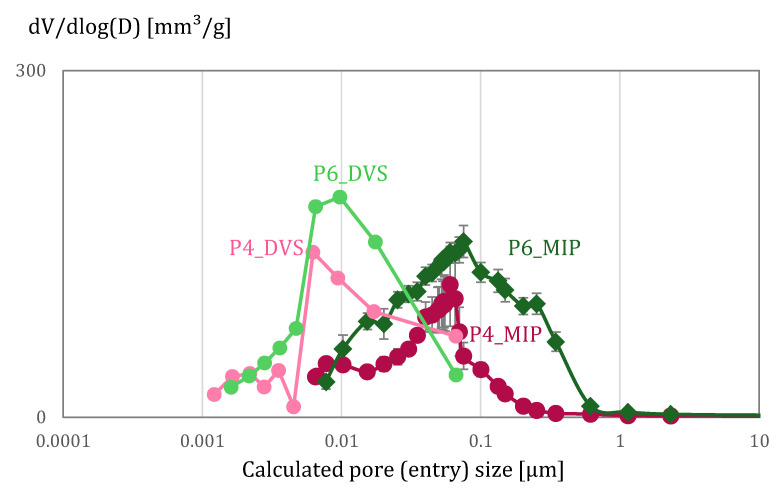
Pore (entry) size distribution of P4 and P6 calculated based on DVS (BJH) and MIP results.

**Figure 7:**
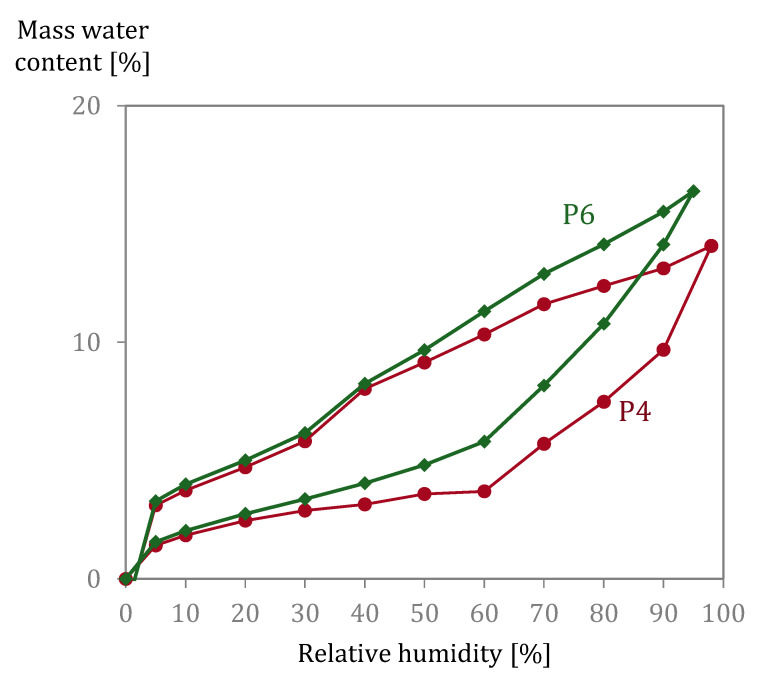
Sorption isotherms (DVS) of P4 (pink rounded markers) and P6 (green rhomboidal markers).

The pore (entry) size distribution calculated from MIP and DVS (BJH + DR) experiments is shown in Figure 6. Differences between results arise on the one hand from the pore size they can measure (as discussed above) and from the presence of the so-called ink-bottle pores. Such pores cause a late mercury intrusion, delayed due to the smallest pore entry sizes to cavities. This derives from a virtual pore size distribution that is finer than the actual pore size distribution. Therefore, for the case of MIP results, they are designated as pore entry sizes. The influence of the presence of ink bottle pores in the DVS results is reflected by the hysteresis seen in sorption/desorption behaviour (Figure 7). Even when comparing the same range of pores measured by MIP and DVS, previous research has found no proportional correspondence [44]. Further discussion on the reasons for the differences obtained with DVS and MIP experiments can be found in [44]. Summarized, it was expected that the accessible volume by the DVS experiment was higher than for MIP measurements. Similar results were measured by [42], and this was also attributed to the differing abilities of these methods to sample different pore sizes.

### 5.3. Deformations during Capillary Imbibition with Water and with Isopropanol

**Figure 8:**
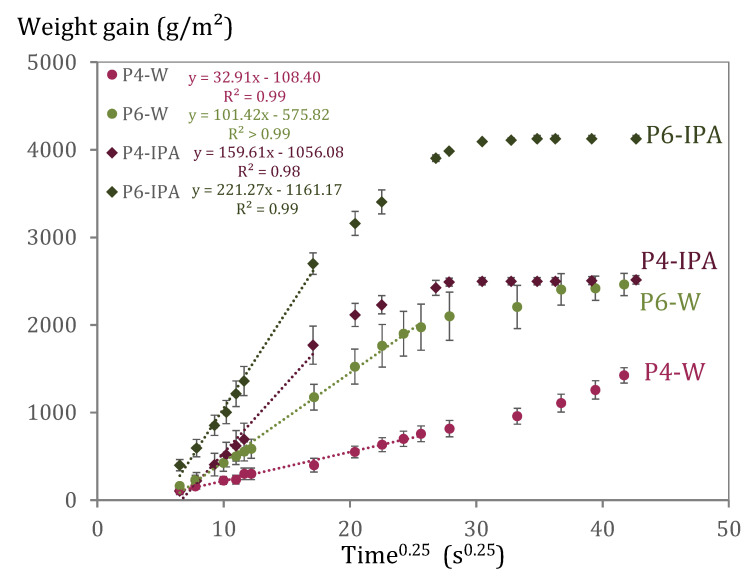
Long-term capillary imbibition performed with water (W) and isopropanol (IPA) in cement pastes P4 and P6. Dotted lines indicate the primary imbibition period (until the liquid reaches the top of the samples). The values of the slopes of the regression curves given above represent the CIR. Dark green and dark pink rhomboidal markers are results of P6 and P4 with IPA as imbibing liquid. Light green and light pink circle markers are results of P6 and P4 with W as imbibing liquid.

Results of capillary imbibition with water and with IPA are shown in Figure 8. The CIRs of the primary period are represented in the graphs as the slope of the least-squares fitting lines (dotted lines) considering the fourth root of time approach [9]. The primary period lasted 26 s^0.25^ and 17 s^0.25^ when testing with water and IPA, respectively. It can be noticed that P6 has a higher CIR than P4, regardless of the used imbibant. This expected result highlights the relevance of the porosity and pore size distribution in the capillary imbibition experiment. The smaller the pore size distribution of P4 (Figure 5) the more likely the presence of restrictive pore sizes, especially considering the localised swelling which contributes to increasing the tortuosity of the pore system. Such swelling is linked to the affinity of C-S-H to water, however, the liquid uptake of the samples in contact with IPA is much larger than the ones with water, due to the different ratio between capillary action and density of the liquid. This is also reflected in the shorter primary imbibition period (until the liquid reaches the top of the samples) for samples exposed to IPA in comparison to samples tested with water. 

After 24 h the IPA uptake is more than seven times larger than the water uptake in terms of volume. Such results indicate that IPA can penetrate the pore structure easier than water. The differences between water and IPA cannot be completely explained by the different capillarity of the paste with IPA and water. IPA has a lower density than water, so the fact that a higher maximum weight gain is obtained with IPA than with water demonstrated that IPA can penetrate a pore size that water cannot access. In addition to the effect of the different surface energy, imbibition is different because both liquids do not interact in the same way with the cementitious matrix. In fact, it was already observed that almost no chemical interaction occurs between the cementitious products and IPA [45,46]. The Report of RILEM TC 238-SCM [24], which compares different hydration stoppage methods, clarifies that from all tested methods (solvent exchange with IPA, vacuum drying, freeze-drying, and oven drying), IPA has the least interactions. Moreover, NMR experiments performed in cement pastes revealed that IPA invades capillary pores that are inaccessible to water [47]. The authors also indicated that the exchange is able to remove free water but has only a minor impact on C-S-H interlayer water. It should be noted that in such experiments, IPA exposure was only 72 h. However, imbibition experiments performed in this research lasted 28 days, so the long-term effects of IPA could have played a role. Nevertheless, results of a higher uptake rate with IPA than with water indicate that IPA moves through the cementitious matrix more easily than water. It should also be considered that IPA can extract the interlayer water causing fine pores to shrink and lead to a coarser pore structure. On the other hand, water can access the amorphous C-S-H gel and make it swell [48]. The difference in the mentioned mechanisms results in easier liquid movement for IPA and hindered liquid movement for water.

The faster penetration of IPA over water seems contradictory with some results of non-aqueous imbibing liquids in the literature. Zeng et al. [49] made capillary imbibition experiments in cement paste using ethanol containing caesium chloride (CsCl) traced by X-ray computed tomography (X-ray CT) and measured the progress of the liquid ingress with time. They also compared their results with those of Yang et al. [50], who performed imbibition experiments in cement pastes with X-ray CT, but with CsCl dissolved in water instead of ethanol. When contrasting the results, Zang et al. [49] found lower rising height and lower ‘absorptivity’ (as the authors call the slope of Δh-√t) with ethanol than with water. This would mean that the liquid ingress rate is lower for ethanol than for water. However, Zeng et al. [49] used a saturated solution of CsCl of 50g/L in ethanol, but the concentration was likely about 7.6 g/L at the most. As the penetration front is measured with the CsCl profile and not with the actual ethanol profile, it is not possible to absolutely confirm that ethanol penetrates more slowly than water. Moreover, Yang et al. [50] used a concentration of 50 g/L CsCl in water, so about ten times higher than the one in ethanol. Considering the coefficients of X-ray mass attenuation for ethanol and CsCl (Figure 2 in [49] at the energy of 160 KeV) the attenuation of the ethanol with the inclusion of this content of CsCl is about only 2.5% lower. So, it is almost the same as doing the X-ray CT with ethanol only, for which the profiling is not very well depicted. Therefore, from such results, the transport behaviour cannot be considered as identical as with pure water. Severe drying can also increase liquid uptake and linearity, while drying was made in both cases at 60 °C, in [49] it lasted 48 h and in [50] the period is unknown since the authors only indicated that it was performed until mass constancy. This might take several weeks depending on the accuracy taken to achieve constant mass and deteriorate the microstructure.

On the other hand, previous research from Hall et al. [51] indicated that the water uptake is consistently lower than results from experiments with other non-aqueous liquids. Additionally, Krus et al. [52] also found a much larger uptake of heptane in comparison to water when testing the absorption of cement pastes. Taylor et al. [53] performed imbibition tests with water and n-decane and also found slower ingress rates in results with water as imbibing liquid. Taylor et al. [53] also attributed this anomaly in Portland cement-based materials to the swelling occurring during the transport process. In general, reported data [51,52,53,54] indicates that absorption results when testing non-aqueous liquids are always larger than when testing with water due to swelling of C-S-H gels [11].

Still, the differences in the properties of the imbibing liquids need to be considered. In this sense, the concept of intrinsic sorptivity has been introduced to describe unsaturated flow by Philip [55], and it aims to describe the capillary imbibition without being influenced by the characteristics of the liquid. This can be approached by considering the intrinsic sorptivity [56] or intrinsic capillary imbibition rate (CIR_int_) as described in (Equation (1)), taking into account the surface tension σ and viscosity η of the imbibing liquid [56]. This is also referred to as F-scaling [57,58], with F being equal to ${\left(\frac{\sigma }{\eta }\right)}^{0.5}$.

(1) $${\mathrm{CIR}}_{\mathrm{int}}=\frac{\mathrm{CIR}}{{\left(\frac{\sigma }{\eta }\right)}^{0.5}}\;$$

**Figure 9:**
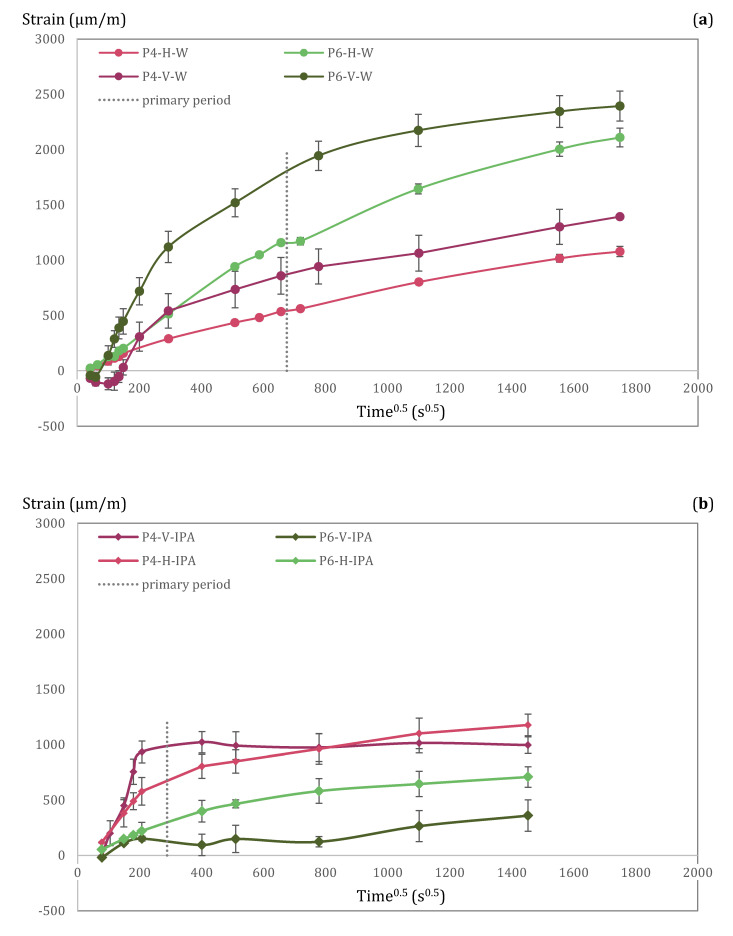
Vertical (V) and horizontal (H) deformations of cement pastes P4 and P6 registered during long-term capillary imbibition with water (**a**) and IPA (**b**) as imbibing liquids.

Even when taking into account the density, the viscosity, and the surface tension of water and IPA, results differ significantly. For the case of water as imbibing liquid, CIR_int_ of P4 and P6 is equal to 3.53 and 10.71 for water and 57.14 and 77.92 for IPA, in [m^0.5^/s^0.25^ × 10^−6^]. This suggests that the anomalously low CIR of cementitious materials can be attributed to their water sensitivity. In that sense, deformations of the pore structure due to swelling play an important role. Horizontal (H) and vertical (V) deformations during capillary imbibition with water and with IPA are shown in Figure 9, demonstrating the different effects of water (a) and IPA (b) on external deformations. Even if there is much more IPA going inside the sample, deformations are rather limited in comparison to the ones registered with water. Moreover, the rate of the deformations suggests that the dynamic microstructural changes are fast enough to affect the imbibition results and cause the lack of linearity on the square root of time. This is consistent with the dependence of the transport phenomenon with the fourth root of time [9,13]. In this sense, McDonald et al. [59] performed ^1^H-NMR measurements in white cement discs during wetting and concluded that pore size changes caused by water ingress are a substantial contributing factor that should be taken into account. Despite the fact that they do not agree that the fourth root of time is optimal to describe imbibition in cementitious materials as supported here, they acknowledge that their own model (under some parameterisations and appropriate timescales) leads to a behaviour that can be well approximated by a t^0.25^ law.

Overall, bulk expansion accompanying capillary water ingress reflects a swelling action of the cementitious matrix. This is very pronounced in the case of contact with water and much less pronounced in the case of IPA. There are still deformations caused by IPA, which can be due to IPA molecules penetrating the C-S-H layers and causing limited swelling [60].

## 6. Discussion

### 6.1. The Thomas and Jennings Model

The TJ model [21] assesses the effect of volumetric variations of the deformable phase (C-S-H) on the pore size depending on the degree of restraint. Clearly, results will be affected by the presence of inert solid phases that enforce a certain degree of restraint. Hence the relative amounts of deformable phases and porosity play major roles. 

Such information is obtained from the relation between the relative fraction of porosity and deformable phases of the system.

(2) $$k_{tot}=k_{p}X_{p}+k_{def}X_{def}+X_{nondef}$$

The deformation of a partially restrained three-phase system is represented by (Equation (2)) [21], where *k_tot_* = ratio of final to initial volumes of the entire composite, *k_p_* = ratio of final to initial volumes of porosity, *k_def_* = ratio of final to initial volumes of the deformable phase, and X denotes the initial volume fraction of a phase (*p*, *def*, and *nondef* for porosity, deformable and non-deformable phases, respectively). A general balance between the volumetric deformations of the system and the deformation of the deforming phase for the particular case in which the initial volume of porosity does not change (*k_p_* = 1) can also be deduced (Equation (3)) [21] when considering that *X_p_* + *X_def_* + *X_nondef_* = 1.

(3) $$k_{tot}=1+X_{def}\left(k_{def}-1\right)$$

Then, a certain threshold or limit value *k_def_*_(*lim*)_ can be obtained for which pores remain the same. If $k_{def}$, is below or above that limit value pores expand or shrink (Equation (4)).

(4) $$<1+\left(\frac{k_{tot}-1}{X_{def}}\right)\;\mathrm{Pores}\;\mathrm{expand}\;\;\;\;\;\;\;\;\;\;\;\;\;\;\;\;\;\;\mathrm{If}\;k_{def}>1+\left(\frac{k_{tot}-1}{X_{def}}\right)\;\mathrm{Pores}\;\mathrm{shrink}\;\;\;\;\;\;\;\;=k_{def\left(lim\right)}=1+\left(\frac{k_{tot}-1}{X_{def}}\right)\;\mathrm{No}\;\mathrm{change}\;\mathrm{in}\;\mathrm{pore}\;\mathrm{volume}$$

**Figure 10:**
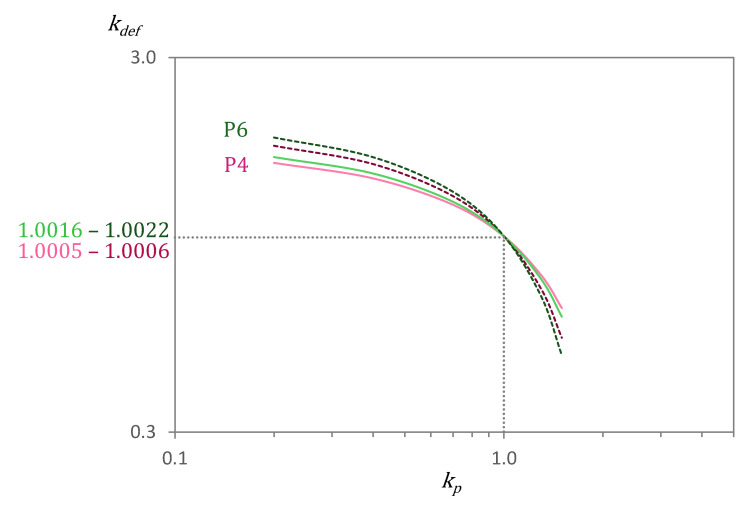
Graphical representation of *k_def_*_(*lim*)_ for cement pastes P4 and P6 after 6 h of capillary imbibition in water (for *k_tot_* = 1.0003 and 1.0009 for P4 and P6, respectively). Full and dotted lines represent the limits of the *k_def_*_(*lim*)_ interval.

The third option in Equation (4) (derived from equations given in [21]) allows determining which is the limit of C-S-H expansion that would result in no changes in the pore volume of the system for a certain external deformation *k_tot_*. If C-S-H expands more or less than such value, then pores either shrink or expand to conform with the external deformation. The determination of such value is of great interest to better understand what occurs inside the cementitious matrix during capillary imbibition. For that, the values of *k_tot_* and *X_def_* need to be known. Deformations measured during capillary imbibition allow the determination of *k_tot_* (final volume/initial volume). We have seen that in fact there are dynamic deformations, so a certain point in time needs to be chosen to determine the deformations at that moment. For this research, we have chosen the deformation values after 6 h of capillary imbibition, as this is a very common experiment duration in the literature. The value of *X_def_*, i.e., the relative amount of C-S-H, was determined in a range of values depending on the method (XRD + PONKCS, DVS and by SEM + PARC, Figure 4). This consequently provides a range of $k_{def\left(lim\right)}$, as it is graphically shown in Figure 10 for the minimum (full lines) and maximum (dotted lines) limits of that range for the case of capillary imbibition after 6 h in water.

The limit value given by *k_def_*_(*lim*)_ (Figure 10) indicates that for a given *k_tot_* there is a value that represents an equilibrium between the total volume change and the change of the deforming phase (i.e., C-S-H swelling) such that the porosity remains the same. This is a simplified model, based on the assumption that the deformable phase is uniform and evenly available to the water. A comprehensive characterization should consider that not all pores might shrink, and some might even expand, leading to different final porosity values. In that sense, changes in porosity due to re-wetting have been observed in NMR studies in cement pastes after 11 days of rewetting [61], where pores larger than 10 nm seemed to reduce whilst pores smaller than 10 nm seemed to increase. This is actually what is proposed by McDonald et al. [59], who interpreted some rearrangement of pore size distribution with water ingress but with the subsequent overall porosity still remaining constant. However, this seems to be a very unique and uncommon case, especially when we consider the strain values registered in the present investigation. 

The *k_def_*_(*lim*)_ values of P4 and P6 for water and IPA indicate that, for the calculated *k_tot_*, the maximum deformations that C-S-H can experience inside the matrix without reducing the porosity are 0.06 and 0.22% for P4 and P6, respectively. The expansion of 34% for C-S-H reported by Taylor [62], when going from dry state equilibrated at 11% RH to the saturated state, are two orders of magnitude larger than such limits, making it very unlikely that C-S-H deformation would be below the *k_def_*_(*lim*)_. This even holds for the case of IPA, where deformations are lower than when testing with water but are still probably causing pore shrinkage. Therefore, results indicate that pores would shrink under the restrictive conditions calculated for the studied cement pastes. Such shrinkage is translated into an increased tortuosity.

### 6.2. Effect of C-S-H Content and Differences in Horizontal and Vertical Deformations

Another interesting point to notice is that the volumetric deformations are not proportional to the C-S-H content. Samples of P6 have a larger porosity than P4 with a similar amount of deformable phase (C-S-H content). As seen in the deformations obtained, the same exposition time to water led to higher deformations in P6 than in P4. This is a relevant point to highlight because it means that the amount of the deformable phase is not directly linked to the magnitude of the volumetric external deformations and the degree of restraint should be considered as well. The relative amount of porosity, non-deformable phases, and C-S-H content all seem to play relevant roles and the anomalous imbibition cannot be solely linked to only one phase. After 28 days of continuous capillary imbibition, volumetric deformations continue to slightly increase and values of P6 are almost double than those of P4. In fact, from the results obtained, three main points can be mentioned: 

(i) Expansion continues for a prolonged time (28 days after the start of the imbibition): NMR studies in mortar samples [63] indicated that the re-invasion of water molecules into the original C-S-H interlayer can be a long-term process that lasts several weeks. Such a time-scale was attributed to the need for water molecules to overcome the strong surface joining forces. Hence, prolonged volumetric deformations are important to describe the long-term behaviour and should be taken into account in the comprehensive modelling of the transport process.

(ii) Similar C-S-H content but with a larger porosity (P6 has 24% more porosity in comparison to P4) leads to a much larger increase in deformations: Even though only two pastes were tested in the present study; it seems that a small relative increase in porosity (for a similar C-S-H content) leads to a significant impact in the external deformations. This can be attributed to the restraints in each mix because with larger porosity, the P6 paste is less restricted and hence external deformations can occur more easily. In other words, swelling of C-S-H translates more into external deformation and less into internal restriction for P6 than for P4. Additionally, it is possible that the unreacted cement would react during imbibition and generate more C-S-H, which is much easier in P6 due to the higher starting porosity. This is not possible for the experiments performed with IPA, where no additional inert phase would be able to react.

(iii) Vertical deformations are larger than horizontal deformations: This indicates that strain results are affected by the direction of the water flow. Vertical deformations appear to be always larger than horizontal deformations. The same difference was also previously reported in mortar and concrete [13]. This might be related to the position of the strain gauge in relation to the water flow (i.e., vertical/horizontal strain gauges placed ‘parallel/perpendicular’ to water flow), but further research is needed to verify this.

The lower deformations in the samples exposed to IPA show the larger sensitivity of cementitious systems to water in terms of the volumetric strains. When a certain liquid is absorbed, an interaction occurs between the adsorbent (cement paste) and the adsorbate (water or IPA). This normally results in a decrease in surface energy and expansion. However, when taking the water sensitivity of cementitious materials into account, generally an anomalously lower water uptake is reported in comparison to other fluids [51,52,53,54]. Then, these differences are also reflected in the deformations measured, as when testing with IPA the registered values are much less in comparison to water. 

### 6.3. Evolution of Deformations with Time

While horizontal deformations are positive during the whole imbibition process, vertical deformations have a different behaviour. When put in contact with water, the immediate expansions at the bottom of the sample lead to a contraction in the vertical axis. Such contraction is reflected as negative values registered by the vertical strain gauges. A schematic representation and further explanation of this effect are described elsewhere [13]. The combination of positive horizontal deformations and negative vertical deformations indicates that the volumetric change corresponds to the effect of the progressive waterfront from the bottom face.

**Figure 11:**
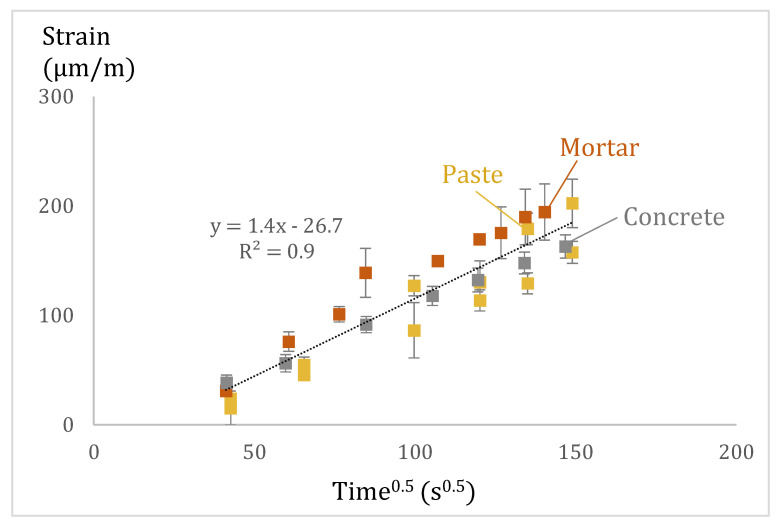
Horizontal strain variation of paste (data from this study), mortar, and concrete (data from [13]) as a function of the square root of time. The dotted line is the linear least-squares fit to the data.

It was also noticed that the evolution of the horizontal deformations in pastes follows a t^0.5^ behaviour during the first 6 h of imbibition, as seen in Figure 9. Interestingly, this seems to occur also in mortar and concrete [13]. Figure 11 shows the strain variation of paste (data from this study, w/b = 0.4 and 0.6), mortar and concrete (data from [13], w/b = 0.45) as a function of the square root of time. Each square data point represents the average of at least three measurements. The dotted line is the linear least-squares fit to the data. It should be mentioned that length changes in mortar and concrete mixes were measured in cylindrical specimens [13] and paste mixes in prismatic specimens. This means that the change in perimeter was measured in mortar and concrete whereas the change in length was measured in pastes. In any case, it is interesting to see that the three series (paste, mortar, and concrete) have a linear relationship with the square root of time during the first 6 h of measurements.

### 6.4. Deformations during the Secondary Imbibition Period

**Figure 12:**
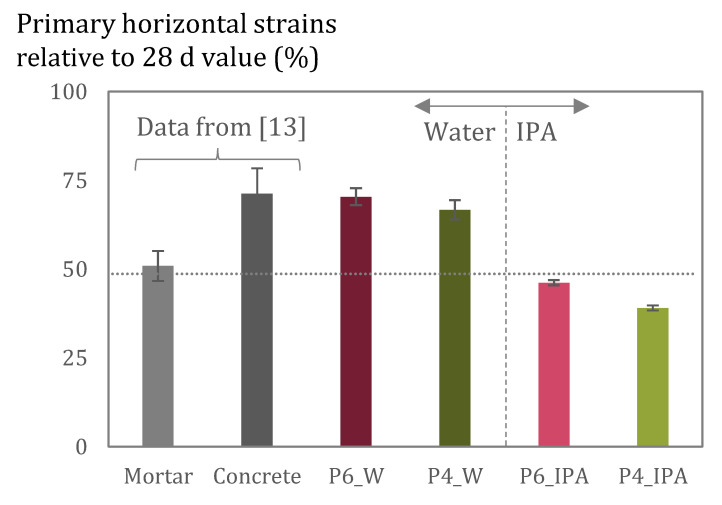
Primary horizontal deformation values relative to strain values at 28 days of exposure for pastes (tested with water and IPA, data from this study), and mortar and concrete (tested with water data from [13]).

Another relevant outcome is the long-term behaviour of pastes during imbibition and the resulting deformations. For the case of the experiments performed with water, most horizontal deformations occur during the primary imbibition period (Figure 12). During this stage, samples deform continuously at a higher rate than during the secondary imbibition period, compatible with the water ingress rate. Between 66 and 70% of the strains (in P4 and P6, respectively) occur during the primary imbibition period (primary deformations), in relation to the deformations measured after 28 days of continuous measurements. As previous research [13] has shown the same behaviour in mortar and concrete samples, results obtained here in pastes confirm that most volumetric changes develop during the primary stage of the water imbibition process for cementitious materials.

Still, there are significant deformations occurring during the secondary period. When analysing the deformations relative to the weight gain, the magnitudes in the secondary period are even larger than during the primary period. Such a trend might be regarded as an indication of the strong interaction between water and the porous cementitious matrix. In that sense, the role of disjoining pressure has been widely discussed in the drying mechanism of cementitious materials [64], and it is probably contributing during water uptake as well. As mentioned, this has also been discussed by Holthausen and Raupach [63] to explain the long-term invasion of water molecules into the original C-S-H interlayer. Although interesting, it is not the aim of this research to investigate the pressure acting inside pores but to investigate the influence of those changes in relation to external deformations. As seen from the results, the evolution of volume change reflected by the strain gauges measurements suggests that pore structure will be highly altered during the primary imbibition period. 

For the case of imbibition experiments with IPA, primary deformations account for 39 and 46%, for P4 and P6, of the deformations registered at 28 days. This means that most deformations seem to occur during the secondary imbibition period. This is linked to the fast uptake that samples experience with IPA, which results in a much shorter primary imbibition period. Even though with IPA most deformations occur during the secondary imbibition period, in absolute terms, they still are smaller than deformations occurring with water.

When considering the amount of deformations reached after 28 days of exposure, paste samples deform more than mortar and concrete samples. The average deformations registered are 355 µm/m and 792 µm/m for concrete and mortar samples, respectively, whereas in paste samples they are 803 µm/m and 1645 µm/m for P4 and P6, respectively. This reflects the level of restraint provided by the presence of aggregates in concrete and mortar in contrast to paste. As concrete and mortar mixes have a higher level of internal restraint, the swelling of the C-S-H is more restricted than in paste and hence fewer deformations occur. Moreover, the content of C-S-H is diluted with the presence of aggregates, reducing the relative amount of deforming phase.

Finally, even though there are relatively less deformations during the secondary imbibition with water, it seems that a sensible amount of deformations still occurs after several weeks. These deformations can no longer be associated with water ingress by capillarity as the secondary period starts only after the main wet front has reached the top of the sample. The continuous water ingress is then connected to the diffusion of water molecules into the laminar structure of C-S-H, and the accompanying deformations seem to be smaller than during the capillary process.

## 7. Conclusions

This research provides a comprehensive description of the anomalous imbibition of cementitious materials in relation to the volumetric variations when testing with water and IPA as imbibants. Results show how considering a rigid pore structure during imbibition provides only a very limited description of the whole transport process. The analysis presented allows the conclusions to be summarised as follows.

- There is volumetric expansion in paste samples during imbibition (certainly when water is the imbibing liquid but to a lesser extend also when IPA is used). The calculations made with the TJ model confirm that the pore structure cannot remain the same after imbibition occurs, but that there is instead some pore shrinkage when the restrictions of the system are considered.
- The direction of the water flow influences the deformations results obtained with strain gauges in different positions. Vertical deformations were always larger than horizontal deformations.
- Results from long-term (28 days) deformation measurements during imbibition in paste reveal that deformations occur as soon as the experiment starts and that they continue growing as time progresses.

Overall, the idea of a rigid pore structure during imbibition seems incomplete and this should be particularly considered in modelling. Moreover, results emphasized the relevance of doing long-term measurements to evaluate the whole transport process.

## Figures and Tables

**Table 1:** Chemical composition of the used cement.

	Wt.%
CaO	63.75
SiO_2_	18.14
Al_2_O_3_	5.28
Fe_2_O_3_	4.18
SO_3_	3.18
CO_2_	1.85
MgO	1.1
K_2_O	0.39
Na_2_O	0.42
TiO_2_	0.34
Mn_2_O_3_	0.1
Insoluble residue	0.74
Loss on ignition	2.48

## Data Availability

The data presented in this study are available upon request from the corresponding author.

## Supplementary Materials

The following are available online at https://www.mdpi.com/article/10.3390/ma15010036/s1. Figure S1: XRD Patterns of P4 and P6 pastes; Figure S2: Phase maps obtained from the SEM/BSE images using the PARC software of P4 (P4_1 and P4_2) and P6 (P6_1 and P6_2) pastes.
