# Supplementary material for: Deformations in Cement Pastes during Capillary Imbibition and Their Relation to Water and Isopropanol as Imbibing Liquids
Source: Materials (Basel). 2021 Dec 21;15(1):36. doi: 10.3390/ma15010036 (PMC8746290; doi:10.3390/ma15010036)
Supplement: Supplementary file 1 [file materials-15-00036-s001.zip › materials-1483993-supplementary.pdf]

Supplementary Materials

Deformations in Cement Pastes during Capillary Imbibition and Their Relation to Water and Isopropanol as Imbibing Liquids

Natalia Mariel Alderete [1,*], Arn Mignon [2], Katrin Schollbach [3] and Yury Villagrán-Zaccardi [1]

[1] Magnel-Vandepitte Laboratory, Department of Structural Engineering and Building Materials, Faculty of Engineering and Architecture, Ghent University, Technologiepark Zwijnaarde 60, B-9052 Ghent, Belgium; yury.villagranzaccardi@ugent.be

[2] Smart Polymeric Biomaterials, Biomaterials and Tissue Engineering Research Group, Campus Group T, KU Leuven, Andreas Vesaliusstraat 13, 3000 Leuven, Belgium; arn.mignon@kuleuven.be

[3] Department of the Built Environment, Eindhoven University of Technology, 5612 AP Eindhoven, The Netherlands; K.Schollbach@tue.nl

* Correspondence: nataliamariel.alderete@ugent.be

XRD diffraction coupled with Rietveld + PONKCS analyses

As mentioned in Section 2 of the research paper, both P4 and P6 XRD patterns are provided below in Figure 1. The PONKCS method addressed the fact that the contribution of partially amorphous phases to the measured diffractogram is proportional to their relative content. The procedure requires a proper model for this contribution and a calibration factor obtained from the pseudo-quantification of a known crystalline phase (i.e. measurements of homogenized samples containing a known amount of ZnO) [22]. The diffuse scattering signal of C-S-H (humps) was fitted using characteristic sets of pseudo Voigt peaks (with their maximums marked as vertical lines in the figures) calibrated from a pure well-hydrated sample.

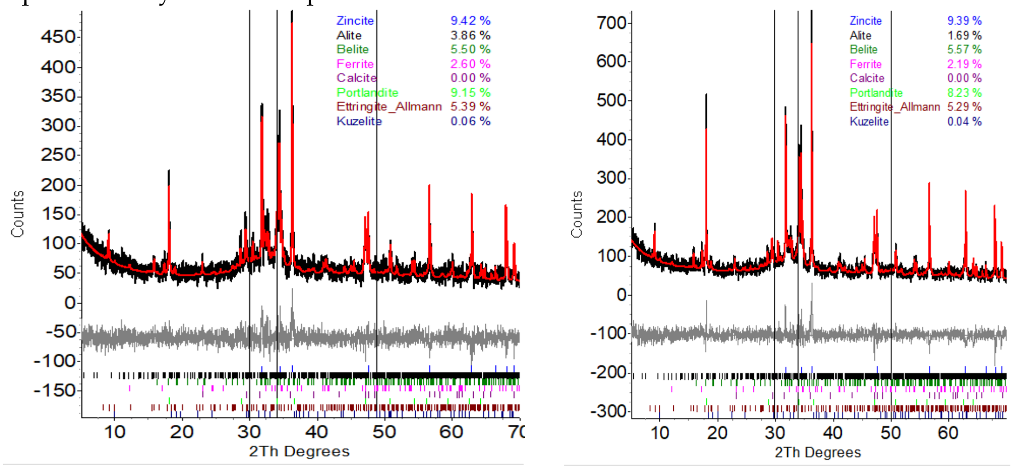

P4 **P6**

Figure S1. XRD Patterns of P4 and P6 pastes.

SEM images with EDS mapping

As mentioned in Section 2 of the research paper, all the phase maps obtained from the SEM/BSE images using the PARC software are provided below in Figure 2. For each individual measurement 3x3 spectral imaging (SI) fields were measured (with one field having a size of 512 pixels x 384 pixels). Each pixel is exactly 1 μm. Since duplicates measurements were made, the total inspected area is 3.54 mm^2. This is a large enough surface to provide representative results for pastes. The SEM/BSE measurements were done with a JEOL JSM-7001F SEM with two 30 mm^2 SDD detectors (Thermo Fisher Scientific) and

NORAN-System7 hardware with NSS.3.3 software. The accelerating voltage was 15 kV with a beam current of 6.2 nA. The measured SI fields were then analysed with PARC software [26, 27]. This software is able to sort the individual pixel spectra into groups based on which element peaks are above a user-defined value. Phase area% are obtained based on the amount of pixel in each group, and can also be represented as a phase map. Phase compositions are based on the quantification of the sum spectra of all pixels from such a group (black = empty spectra, light green = C-S-H, dark green = Aluminate, light blue = C_2S, dark blue = C_3S, grey = portlandite).

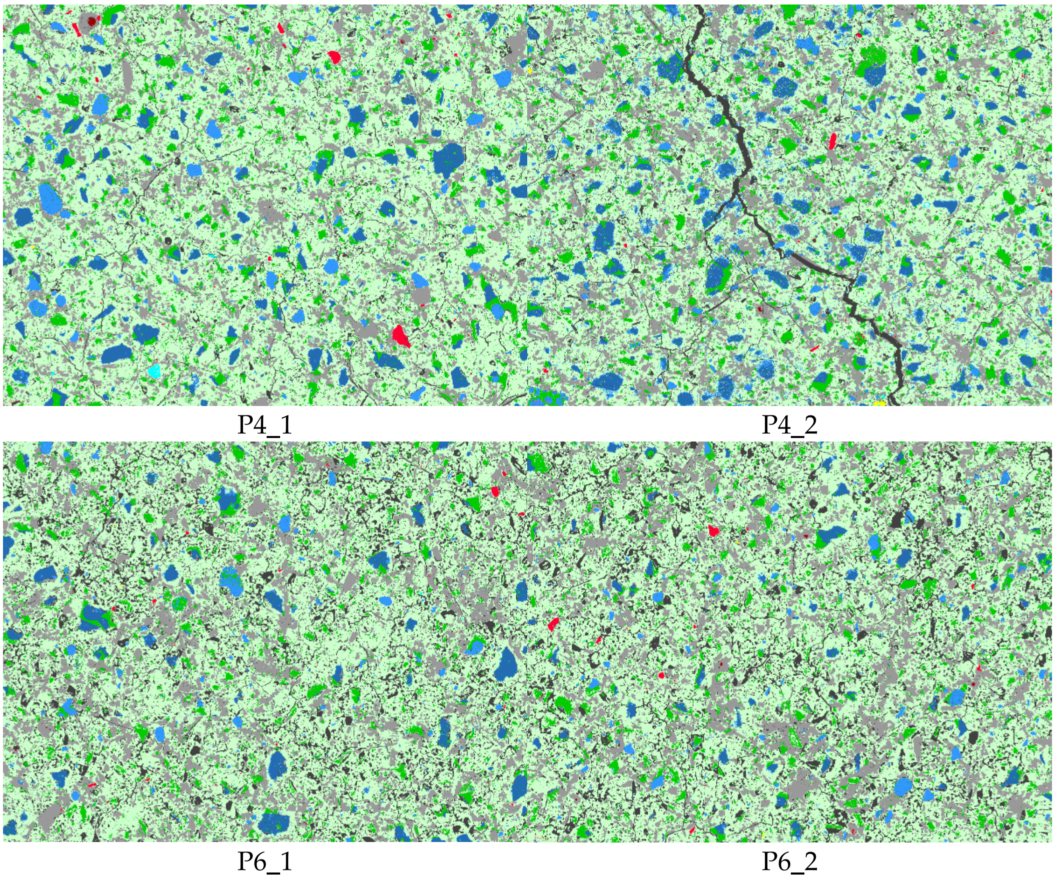

Figure S2. Phase maps obtained from the SEM/BSE images using the PARC software of P4 (P4_1 and P4_2) and P6 (P6_1 and P6_2) pastes.
